# Analysis of *IL28B* Variants in an Egyptian Population Defines the 20 Kilobases Minimal Region Involved in Spontaneous Clearance of Hepatitis C Virus

**DOI:** 10.1371/journal.pone.0038578

**Published:** 2012-06-14

**Authors:** Vincent Pedergnana, Mohamed Abdel-Hamid, Julien Guergnon, Amira Mohsen, Lénaïg Le Fouler, Ioannis Theodorou, Mostafa Kamal Mohamed, Arnaud Fontanet, Sabine Plancoulaine, Laurent Abel

**Affiliations:** 1 Laboratory of Human Genetics of Infectious Diseases, Necker Branch, Institut National de la Santé et de la Recherche Médicale U980, Paris, France; 2 University Paris Descartes, Paris, France; 3 Viral Hepatitis Research Laboratory, National Hepatology and Tropical Medicine Research Institute, Cairo, Egypt; 4 Department of Microbiology, Minia Faculty of Medicine, Minia, Egypt; 5 Laboratory of Immunity and Infection, Institut National de la Santé et de la Recherche Médicale UMR-S 945, University Pierre et Marie Curie Paris 6, Groupe Hospitalier Pitié-Salpêtrière AP-HP, Paris, France; 6 Department of Community, Environmental and Occupational Medicine, Faculty of Medicine, Ain Shams University, Cairo, Egypt; 7 Institut Pasteur, Unité d’Epidémiologie des Maladies Emergentes, Paris, France; 8 Conservatoire National des Arts et Métiers, Chaire Santé et Développement, Paris, France; 9 St. Giles Laboratory of Human Genetics of Infectious Diseases, Rockefeller Branch, The Rockefeller University, New York, New York, United States of America; Inserm, U1052, UMR 5286, France

## Abstract

Spontaneous clearance of hepatitis C virus (HCV) occurs in ∼30% of acute infections. Host genetics play a major role in HCV clearance, with a strong effect of single nucleotide polymorphisms (SNPs) of the *IL28B* gene already found in different populations, mostly infected with viral genotypes 1 and 3. Egypt has the highest prevalence of HCV infection in the world, which is mostly due to viral genotype 4. We investigated the role of several *IL28B* SNPs in HCV spontaneous clearance in an Egyptian population. We selected nine SNPs within the *IL28B* genomic region covering the linkage disequilibrium (LD) block known to be associated with HCV clearance in European populations. These SNPs were genotyped in 261 HCV-infected Egyptian subjects (130 with spontaneous clearance and 131 with chronic infection). The most associated SNPs were rs12979860 (P = 1.6×10^−7^) and the non-synonymous *IL28B* SNP, rs8103142 (P = 1.6×10^−7^). Interestingly, three SNPs at the two bounds of the region were monomorphic, reducing the size of the LD block in which the causal variants are potentially located to ∼20 kilobases. HCV clearance in Egypt was associated with a region of *IL28B* smaller than that identified in European populations, and involved the non-synonymous *IL28B* SNP, rs8103142.

## Introduction

Hepatitis C virus (HCV) infects 170 million people worldwide and is thus a major Public Health problem [1], [2]. End-stage chronic hepatitis C is the leading cause of liver transplantation in developed countries and more than 350,000 people die from HCV-related liver diseases each year [1], [2]. Spontaneous clearance of hepatitis C virus (HCV) occurs in ∼30% of acute infections [3], [4]. Differences in HCV clearance and persistence have been associated with viral factors and several host factors, including ethnicity, age, sex and co-infection with human immunodeficiency virus or hepatitis B virus [4], [5]. Host genetics also play a major role, with a strong effect of variants of the *IL28B* gene, which encodes IFN-λ3, on spontaneous HCV clearance reported in populations of different backgrounds, mostly infected with viral genotypes 1 and 3 (reviewed in [3]). The same *IL28B* variants are also strongly associated with HCV clearance under treatment [6], [7].

Egypt has the highest prevalence of HCV infection of any country in the world, with a seroprevalence ranging from 10% in children to 45% in adults [8], [9]. More than 90% of HCV infections in Egypt involve genotype 4 [8], [9]. One study recently reported an association between the most studied single nucleotide polymorphism (SNP) of *IL28B*, rs12979860, and the spontaneous clearance of HCV genotype 4 in an Egyptian sample [10]. We investigated the role of a panel of *IL28B* SNPs in the spontaneous clearance of HCV in a larger Egyptian cohort. We found that the clearance effect was restricted to a region of *IL28B* smaller than that identified in European populations and that this effect also involved rs8103142, a non synonymous *IL28B* SNP.

## Methods

This study combined data from two Egyptian cohorts. The first included 3994 individuals from a large epidemiological study in a village of the Nile delta, the details of which have been described elsewhere [11]. Briefly, in this cohort 491 individuals (12.3%) were seropositive for HCV and 305 of them had positive PCR test. None of the seropositive individuals have been treated at the time of the study. Ninety-six RNA samples had previously been sequenced in this cohort, and 95 (99%) of these samples were of HCV genotype 4 [11]. From the overall cohort, we selected 188 independent unrelated HCV-infected subjects defined on the basis of two positive serological tests [11]. Individuals with positive PCR test for viral RNA were further classified as “chronically infected”, whereas those with negative results were considered to have cleared the virus spontaneously. Subjects of this cohort were not tested for HBV infection, but it could be noted that several studies in Nile Delta villages, including this one, reported a rather low prevalence on HBV infection (2%–8%) [12], [13], [14]. The second cohort included 73 patients with symptomatic acute HCV infection recruited from Cairo hospitals in the context of a study that has been described elsewhere [15], [16]. In these subjects, spontaneous clearance was defined as the loss of HCV RNA from the serum in the absence of treatment, based on two consecutive negative viral PCR results, together with subsequent negative tests, including at least one more than one year after the acute episode. Chronic infection was defined as the presence of HCV RNA in the serum in all PCR tests over a period of at least one year after the acute episode. Twenty-seven RNA samples from this cohort had previously been sequenced, and 24 (89%) were of HCV genotype 4 [15], [16]. Subjects of this cohort were tested for HBV infection, as previously described [17], and out of the 73 present patients, only one was found to be HBV infected. Finally, the overall sample of 261 subjects consisted of 130 subjects with spontaneous clearance and 131 with chronic infection. All these subjects were not tested for HIV infection, but HIV seroprevalence was found to be quite low (<0.1%) in the general Egyptian population aged of 15 to 49 years (http://www.unaids.org/en/regionscountries/countries/egypt) as well as among injection drug users (∼0.6%) [18]. The study was approved by the Institutional Review Board of the Egyptian Ministry of Population and Health. Signed informed-consent documents were obtained from all study participants before they entered the study.

**Figure 1:**
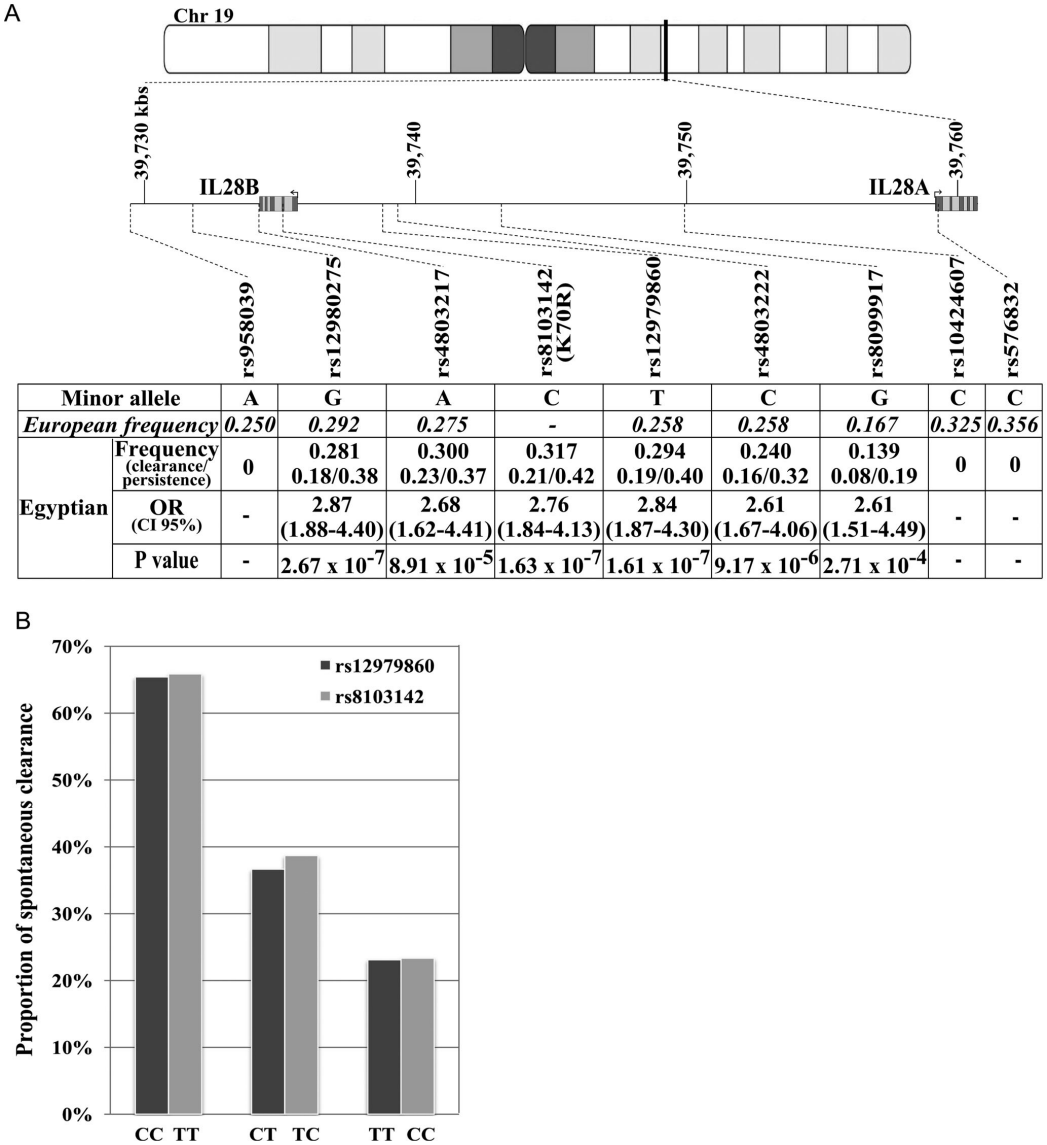
SNPs genotyped in the IL28B genomic region and their association with spontaneous clearance. A. The top panel shows the IL28B genomic region bounded by rs958039 and rs576832, its position on chromosome 19 (39,730,301–39,759,282 base pairs), and the respective positions of the genotyped SNPs. The bottom panel shows the frequencies of the minor allele for each SNP in the European and Egyptian populations. European frequencies are estimated from the CEU data from Hapmap and the 1000 Genomes project. Egyptian frequencies are estimated from the population from the overall sample studied here and separately for the two groups of individuals (HCV clearance and HCV persistence). Odds ratio for HCV clearance (OR) and 95% confidence intervals (CI 95%), together with P-values for univariate tests with the additive genetic model are also shown for each SNP. B. Proportion of spontaneous clearance as a function of genotype at SNPs rs12979860 and rs8103142.

We genotyped a total of nine SNPs within the *IL28B* genomic region (Figure 1A). We first included the three SNPs (rs12980275, rs12979860, rs8099917) most frequently reported to be associated with HCV clearance in previous studies. We then explored the long-range linkage disequilibrium (LD) (500 kilobases on either side of *IL28B*) of these three SNPs, using the European CEU population available data from HapMap (http://www.hapmap.org) and 1000 Genomes project [19] databases. We selected the two most distant SNPs (rs958039, rs576832) in LD, with an r^2^>0.3, with any of the three initial SNPs. Within the chromosome 19 region defined by these two SNPs, we included three additional regularly spaced SNPs (rs4803217, rs4803222, rs10424607) in LD, with an r^2^>0.3, with any of the three initial SNPs. Finally, we also selected the non synonymous SNP rs8103142 not present in the public databases but reported to be in strong LD with rs12979860 in European populations [20]. Illumina GoldenGate genotyping was carried out with VeraCode technology (Illumina). For genetic markers, we applied the following quality control criteria: call rate <90%, Hardy-Weinberg *P*-value<0.005 or low-quality genotype clustering. No individuals were excluded on the basis of genotyping call rate.

Univariate tests of association between the genotyped SNPs and the clearance/persistence status of HCV infection were carried out using PLINK software [21]. Multivariate analysis was performed with the LOGISTIC procedure implemented in SAS software v.9.2 (SAS Institute, Cary, North Carolina, USA). Pairwise LD between SNPs and haplotype frequencies were estimated with PLINK [21] and Haploview 4.1 (http://www.broad.mit.edu/mpg/haploview) software.

## Results

All the SNPs studied satisfied the quality control filters in all 261 individuals, and the allelic frequencies are reported in figure 1A. Three SNPs at the 3′ (rs958039) and the 5′ (rs10424607 and rs576832) boundaries of the *IL28B* region were monomorphic, whereas the frequencies of the other SNPs were similar to those observed in European populations. Univariate association analyses showed that the six polymorphic SNPs in the Egyptian population were strongly associated with spontaneous HCV clearance (Figure 1A). The most significant results were obtained with an additive genetic model, with rs12979860 [odds ratio of presenting HCV clearance (OR) for CT vs.TT (or CC vs.CT) = 2.84 (95% confidence interval: 1.87–4.30); P = 1.6×10^−7^] and rs8103142 [OR = 2.76 (1.84–4.13); P = 1.6×10^−7^]. Association analyses conducted for these two SNPs separately in our two cohorts provided very similar results in terms of allele frequencies and odds-ratios indicating that possible HBV co-infection in some patients of our first cohort could not affect these results. In our whole sample, and taking rs8103142 as an example, the proportion of subjects with HCV clearance was much higher in subjects carrying the protective TT genotype (66% of clearers) than in those with CT (39% of clearers) or CC (23% of clearers) genotypes (Figure 1B).

We then carried out multivariate regression analysis with the six polymorphic SNPs. When the most significant SNP (rs12979860) was introduced into the model, the other five SNPs ceased to be significantly associated with clearance, confirming that this association was based on a single signal. We then estimated pairwise LD between the six polymorphic SNPs. The LD pattern of these SNPs, and that of rs12979860 in particular (see Figure S1A), in this Egyptian population was similar to that in European populations (see Figure S1B). The non-synonymous SNP rs8103142 could not be included in this comparison, as no genotyping data were available for the CEU population. The r^2^ for the relationship between rs8103142 and rs12979860 was estimated at 0.96 in another European population [20] and 0.88 in our Egyptian population. The three remaining SNPs were monomorphic in the Egyptian population, whereas they form part of the *IL28B* cluster associated with HCV clearance in the European population (Figure 1A). Interestingly, these three SNPs are located at the 3′ (rs958039) and 5′ (rs10424607 and rs576832) ends of the genomic region corresponding to *IL28B* (Figure 1A), reducing the size of the LD block in the Egyptian population by about one third. Overall, these observations suggest that the causal SNPs underlying the association with HCV clearance are located in a smaller region of ∼20 kilobases located between rs958039 and rs10424607 rather than in a long-range LD block.

**Figure 2:**
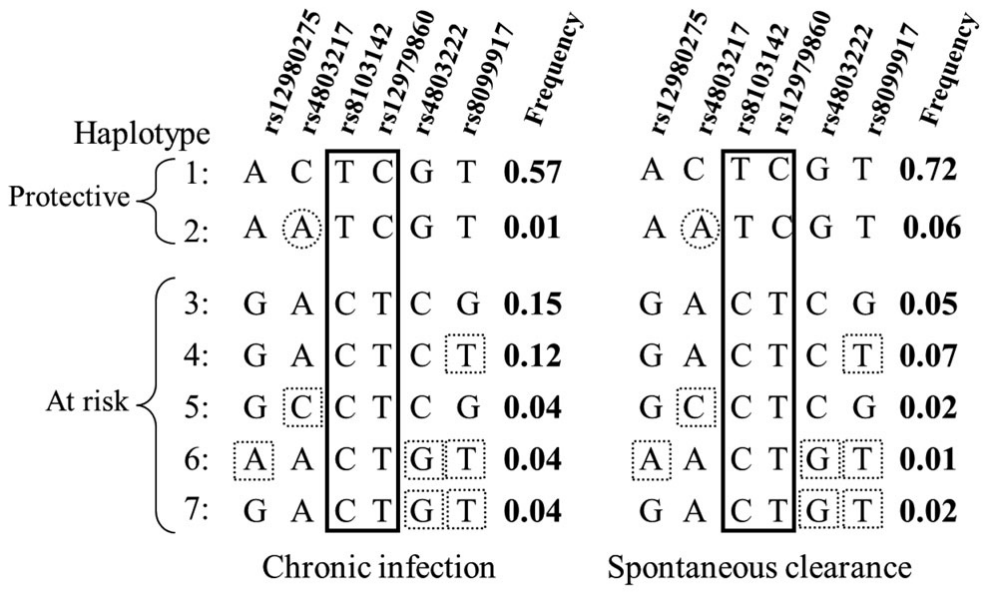
Haplotype frequencies estimated from the Egyptian population, as a function of HCV status. Haplotype frequencies are given for each of the two groups: individuals with persistent infection (“Chronic infection”) and individuals who cleared the infection (“Spontaneous clearance”). As defined in the text, the protective haplotypes (1 and 2) favour HCV clearance, whereas the “at risk” haplotypes (3 to 7) confer a predisposition to chronic HCV infection. The more frequent protective haplotype (haplotype 1) could be defined as the reference protective haplotype, while the more frequent “at risk” haplotype (haplotype 3), which is composed of the exact opposite alleles for all SNPs of haplotype 1, could be defined as the reference “at risk” haplotype. The dashed-line circles show discrepancies within the two protective haplotypes, i.e. between haplotype 2 and the reference protective haplotype 1. The dashed-line squares show discrepancies within the “at risk” haplotypes, i.e. between haplotypes 4–7 and the reference “at risk” haplotype 3. Only two SNPs identified by the solid-line rectangle (rs8103142 and rs12979860) shows have the same allele for the two protective haplotypes and the alternative allele in all the five at risk haplotypes.

Finally, we estimated haplotype frequencies derived from the six polymorphic SNPs in this population. The objective of this haplotype analysis was not to test formally for differences in haplotype frequencies between subjects with clearance and those with chronic infection, but to further refine our univariate results by providing the general haplotype structure of this Egyptian sample. Seven haplotypes had an estimated frequency >0.02 in the whole sample. Based on differences in haplotype frequencies between subjects with spontaneous clearance and those with chronic infection, these seven haplotypes could be classified into two protective (favouring clearance) haplotypes (one of which was much more common than the other) and five “at risk” haplotypes (conferring a predisposition to chronic infection) (Figure 2). The two protective haplotypes differed only in terms of the allele of SNP rs4803217. In comparisons with the alleles present in the two protective haplotypes, only two SNPs presented the alternative allele in all five at risk haplotypes, discrepancies being observed for the others. The two SNPs with consistently different alleles between the protective and at risk haplotypes were rs12979860 and rs8103142, the non-synonymous SNP. This observation is consistent with the univariate association results and supports the view that the core of the association is dependent on these two SNPs, the effect of which are indistinguishable in this sample.

## Discussion

These results confirm the major role of *IL28B* variants in the spontaneous clearance of HCV genotype 4 infection in an Egyptian population. In particular, the magnitude of the association with rs12979860 observed is similar to that reported in many studies conducted on HCV genotype 1 infection [5], [20] and with the only previous study of HCV genotype 4 infection in an Egyptian population [10]. Our refined analysis, including eight additional *IL28B* SNPs, showed that the LD block of the *IL28B* SNP cluster associated with HCV clearance was shorter than that in European populations. In particular, this analysis reduces the chromosomal region in which the causal SNPs are potentially located to about 20 kilobases of the *IL28B* genomic region, excluding the *IL28A* genomic region. Finally, both our univariate and haplotype analyses clearly showed that the core association depended on two SNPs, rs12979860 and rs8103142. SNP rs8103142 replaces a lysine residue by an arginine residue at position 70 (K70R) in IFN-λ3, a substitution predicted to be benign by Polyphen [22]. Further functional studies are required to identify the causal SNPs underlying this strong association and the precise mechanisms of *IL28B* involvement in HCV infection.

## Supporting Information

Figure S1 **Linkage disequilibrium pattern for the six polymorphic SNPs in the Egyptian and European populations.** Panel A shows linkage disequilibrium (LD) in terms of r^2^ values between rs12979860 and the eight other genotyped SNPs in two different populations (Egyptian/European). European r^2^ values were estimated from the CEU data from Hapmap and the 1000 Genomes project, Egyptian values were estimated from our overall sample. No data for rs8103142 were available for the CEU population, so the r^2^ value for this SNP could not be estimated for the European population. The SNPs rs955155, rs10424607 and rs576832 were monomorphic in the Egyptian population, and it was therefore not possible to estimate r^2^ values for these SNPs in the Egyptian population. Panel B shows the pairwise r^2^ for combinations of the six polymorphic SNPs of the *IL28B* genomic region in both the Egyptian population (first value) and the CEU European population (second value). No data for rs8103142 were available for the CEU population. Differences of less than 10% between the two r^2^ values are indicated in dark grey; differences of less than 25% are shown in light grey and differences of more than 25% are shown in white.
(DOC)
