# Supplementary figures and images for: Analysis of IL28B Variants in an Egyptian Population Defines the 20 Kilobases Minimal Region Involved in Spontaneous Clearance of Hepatitis C Virus
Source: PLoS One. 2012 Jun 14;7(6):e38578. doi: 10.1371/journal.pone.0038578 (PMC3375300; doi:10.1371/journal.pone.0038578)

**Supporting Information**

**Supplementary Figure S1**


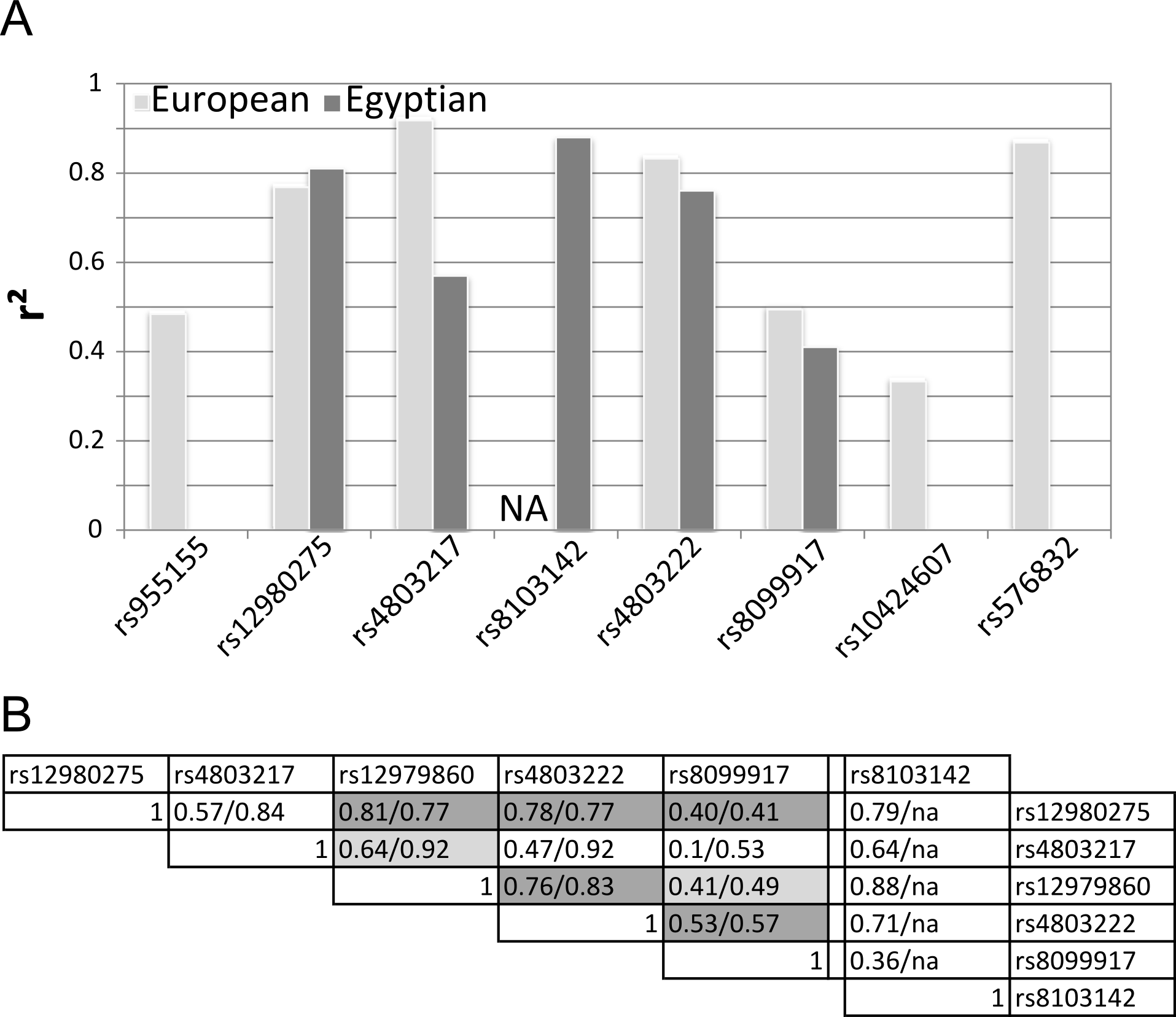

Supplement: Figure S1 — Linkage disequilibrium pattern for the six polymorphic SNPs in the Egyptian and European populations. Panel A shows linkage disequilibrium (LD) in terms of r2 values between rs12979860 and the eight other genotyped SNPs in two different populations (Egyptian/European). European r2 values were estimated from the CEU data from Hapmap and the 1000 Genomes project, Egyptian values were estimated from our overall sample. No data for rs8103142 were available for the CEU population, so the r2 value for this SNP could not be estimated for the European population. The SNPs rs955155, rs10424607 and rs576832 were monomorphic in the Egyptian population, and it was therefore not possible to estimate r2 values for these SNPs in the Egyptian population. Panel B shows the pairwise r2 for combinations of the six polymorphic SNPs of the IL28B genomic region in both the Egyptian population (first value) and the CEU European population (second value). No data for rs8103142 were available for the CEU population. Differences of less than 10% between the two r2 values are indicated in dark grey; differences of less than 25% are shown in light grey and differences of more than 25% are shown in white. (DOC) [file pone.0038578.s001.doc]
